# Shape facilitates number: brain potentials and microstates reveal the interplay between shape and numerosity in human vision

**DOI:** 10.1038/s41598-020-68788-4

**Published:** 2020-07-24

**Authors:** Elena Gheorghiu, Benjamin R. Dering

**Affiliations:** Department of Psychology, University of Stirling, Stirling, FK9 4LA Scotland UK

**Keywords:** Cognitive neuroscience, Perception, Electroencephalography - EEG, Psychophysics

## Abstract

Recognition of simple shapes and numerosity estimation for small quantities are often studied independently of each other, but we know that these processes are both rapid and accurate, suggesting that they may be mediated by common neural mechanisms. Here we address this issue by examining how spatial configuration, shape complexity, and luminance polarity of elements affect numerosity estimation. We directly compared the Event Related Potential (ERP) time-course for numerosity estimation under shape and random configurations and found a larger N2 component for shape over lateral-occipital electrodes (250–400 ms), which also increased with higher numbers. We identified a Left Mid Frontal (LMF; 400–650 ms) component over left-lateralised medial frontal sites that specifically separated low and high numbers of elements, irrespective of their spatial configuration. Different luminance-polarities increased N2 amplitude only, suggesting that shape but not numerosity is selective to polarity. Functional microstates confined numerosity to a strict topographic distribution occurring within the LMF time-window, while a microstate responding only to shape-configuration was evidenced earlier, in the N2 time-window. We conclude that shape-coding precedes numerosity estimation, which can be improved when the number of elements and shape vertices are matched. Thus, numerosity estimation around the subitizing range is facilitated by a shape-template matching process.

## Introduction

Numbers rule our lives: we possess a strong visual sense of number that is used to rank, estimate, and quantify everything we see in the environment (e.g., a crowd on the street, number of students in a lecture hall). We can estimate numerosities over a broad range, between one to hundreds or thousands of items, without involving cognitive processes such as counting which are ineffective for short viewing times^1^. For larger numbers, numerosity perception is subserved by mechanisms activated by different ranges of numerosity and/or density^2,3^. Numerosity estimation is fast and accurate when the number of items is less than four which has led to the concept of subitizing^4–7^, with larger numbers between 5 and 7 being estimated rapidly but with larger errors^8^ (but see^6,7,9–15^ for a subitizing limit of up to 7 elements). Numerosity estimation may also be linked to geometric cues in the formation of an object’s shape as demonstrated with dice dot-patterns^12,16,17^, which suggests that processing of small quantities might be linked to high-level pattern recognition (e.g., shape-template matching). Similarly, shape detection studies have found that simple shapes (e.g., triangle, square, pentagon) are detected faster and more accurately than complex shapes containing more vertices^18^.

To encode the shape of an object, the visual system must spatially integrate information about the relationship between different parts along the shape outline^18,19^. For example, numerous studies have examined shape processing using radial frequency (RF) patterns, which are created by sinusoidally modulating the radius of a circle, with the number of full cycles of modulation per 2π radians being the RF number (e.g., triangles/RF3, squares/RF4, and pentagons/RF5)^19–24^. Global RF shape processing is thought to depend upon pooling together information about the points of maximum curvature, i.e., the peaks and troughs (vertices/corners) of the RF pattern^19,25–30^. Loffler et al.^18^ examined the contribution of local (e.g., orientation and position of segments) and global processing, by exploring *the amount* of global information pooling in a RF shape discrimination task and showed that global pooling only extends up to about RF5 shape (pentagon), while higher frequency RF shapes rely mainly on local processing. Given that global shape processing is limited by the number of shape vertices^18^, which overlaps with proposed subitizing limits^8,10^, it raises the question as to whether numerosity perception and shape processing are intrinsically linked, by sharing a common neural mechanism.

In addition, previous studies have shown that luminance polarity alternation (that is, an inconsistency in the luminance polarity of consecutive elements that make up a shape) disrupts the processing of RF patterns^31,32^, long straight lines^33^ and curvature^34^. For example, RF detection thresholds increase and shape aftereffects are reduced when the number of consecutive elements of the same polarity decrease^31^. These findings suggest that changes in luminance polarity negatively affects shape processing mechanisms, and it distorts the perception of shape itself^31,32^. Since we asked whether shape processing and numerosity estimation share a common neural mechanism, it is likely that any shared neural mechanism would also be affected by changes in luminance polarity. In this communication, we address these issues by using behavioural measures (reaction time and accuracy), Event Related Potentials (ERPs) and functional microstates to map the time-course of the interplay between shape configuration and luminance polarity on numerosity estimation within and nearby the subitizing range.

ERP studies of subitizing have highlighted the early N1 component as a possible candidate for numerosity estimation, with N1 amplitudes increasing with numerical quantities^35–38^. Some studies have shown the N1 amplitude plateaus for 3 elements^39,40^, yet others suggest a higher quantity^9,35^. Nan et al.^9^ showed that the amplitude of the N1 component is maximal for high numerosities, asymptotes when the number of elements is above 8, and indexes all elements in the stimuli irrespective of these being targets or distractors. As for specifically estimating number, two ERP components have been identified—the N2pc and CDA, or Contralateral Delayed Activity—for a review see^41^. First, the N2pc is a difference wave reflecting inter-hemispheric differences observed between 170 and 300 ms for target stimuli presented in the left and right visual hemi-fields^42,43^. The N2pc effect is reportedly maximal for stimuli containing 3–4 elements, individuating low numeric quantities^43,44^. Second, the CDA, a lateralized effect like N2pc, while also modulated by target quantity, ostensibly reflects the encoding and maintenance of items in working memory^45,46^. Importantly, these studies used visual features of the elements (e.g., color) to segregate targets from distractors for numerosity estimation. This implies that the generation of N2pc and CDA are explicitly linked to feature-based attention mechanisms. Therefore, in our experiments we also examine the effect of stimulus features, such as spatial configuration and luminance polarity (black vs. white) on numerosity estimation, without the demand from feature and location-based attention.

**Figure 1:**
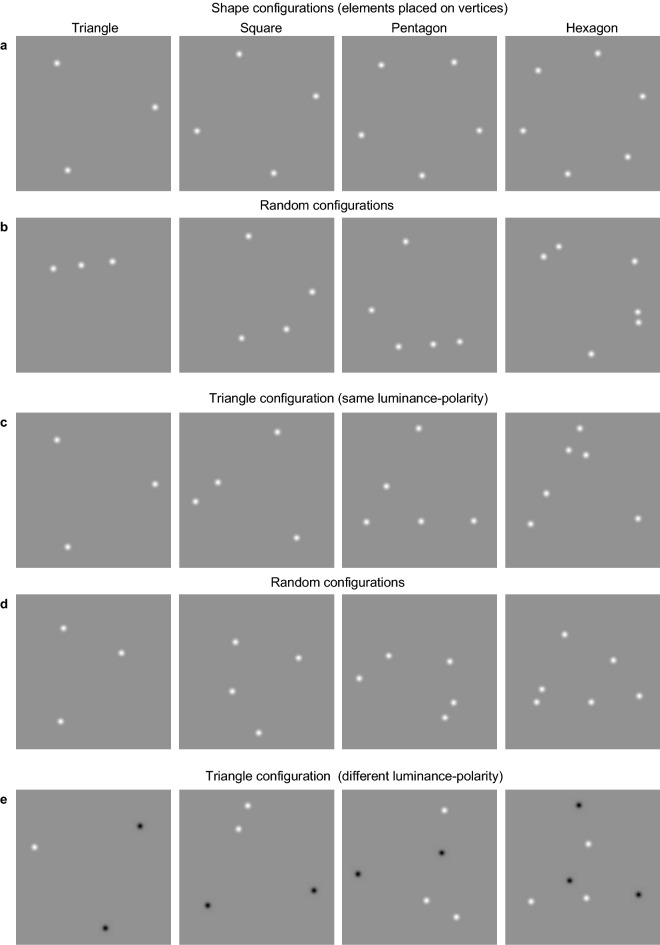
Experimental stimuli and procedure. Stimuli consisted of a small number of elements, either 3, 4, 5 or 6. The elements were positioned either **(a)** on the vertices of simple shapes, e.g., equilateral triangle, square, pentagons and hexagon or **(b)** randomly. **(c)** Equilateral triangle configuration sampled by either 3, 4, 5 or 6 elements, with three elements always positioned on the vertices and the remaining elements placed randomly anywhere within and including the virtual contour-path. **(d)** random only condition, which is the same as (c) except that no elements were placed on the vertices. **(e)** Stimuli made of different (white and black) luminance-polarity elements, with three elements always positioned on the vertices of a triangle and the remaining elements placed randomly anywhere within and including the virtual contour-path. Examples of same luminance-polarity (all white or all black) condition used in Experiment 3 is shown in **(c)**. For all experiments, the orientation of each virtual shape configuration was randomized from trial to trial.

Here, using behavioural measures (reaction times and accuracy), ERPs, and functional microstates, we examined whether spatial configuration and its complexity (i.e., the number of shape vertices) facilitate numerosity estimation. Stimuli consisted of a small number of elements (3, 4, 5 or 6) positioned either on the vertices of simple shapes (e.g., equilateral triangle, square, pentagon and hexagon; Fig. 1a) or randomly, anywhere on the virtual contour-path of a shape, except the vertices (or corners) (Fig. 1b). For this experiment (Experiment 1), the number of elements and shape vertices were matched i.e., three elements placed on the vertices of a triangle, and so on (Fig. 1b; see “Methods”). Stimuli were presented for 100 ms only, in order to avoid counting, and the participant’s task was to indicate as quickly and as accurately as possible the number of elements perceived on the screen by pressing the corresponding key. We predicted that shape configuration would facilitate the ERP response to numerosity, since there is evidence that early ERP components of visual perception (N1, N2) are enhanced in amplitude by the perception of illusory contours forming Kanizsa squares^47^. Our virtual shape configurations by their very nature resemble a collection of illusory lines that are integrated into shapes (although without any perception of illusory boundary between elements as in the case of Kanizsa figures). Hence, we expected to find higher N1 and N2 amplitudes for shape versus random configurations, but note that the mechanisms of illusory shape perception and shape coding in our experiments, which use Gaussian elements, are different.

In Experiment 2, we dissociated shape complexity from numerosity by utilizing the least complex shape (a triangle) sampled by the full range of elements, in comparison to random configurations. Triangular stimuli were sampled by either 3, 4, 5 or 6 elements, with three elements always positioned on the vertices and the remaining elements placed randomly anywhere within and including the virtual contour-path (Fig. 1c). Triangle conditions were compared with their corresponding random conditions, which were the same as the triangle configuration, except that none of the elements were placed on vertices. Based on numerosity studies involving feature-based attention to individuate numbers (for a review see Mazza and Caramazza^41^) which highlight N1, N2pc and CDA components, we could predict an N1, N2 and a late component that all respond preferentially to low numeric quantities (3 or 4 elements). Furthermore, microstate segmentation would allow us to separate the functionality of these three ERP components in relation to shape and numerosity coding. Specifically, we expected to find distinct microstates overlapping the time-course of our predicted ERP components (N1, N2, late component). In addition, microstate analysis allows us to establish whether these ERPs result from distinct neural source generators that encode numerosity, and their time-course, or utilise the same neural source generators for numerosity and shape processing.

Finally, given that previous studies of shape processing have shown that luminance polarity distorts shape perception^31–33^, we examined whether numerosity mechanisms are selective to elements’ luminance polarity (Experiment 3) by comparing stimuli in which all elements have either the same (all white or all black) or different luminance polarity (white and black). We predicted better performance in the same compared to different polarity conditions, suggesting that luminance polarity consistency can facilitate numerosity estimation in a similar fashion to shape configurations. We expected to find that early ERP components reflecting low-level visual feature processing would be sensitive to different polarities, which may in turn disrupt numerosity estimation.

## Results

### Effect of spatial configuration (shape vs. random)

**Figure 2:**
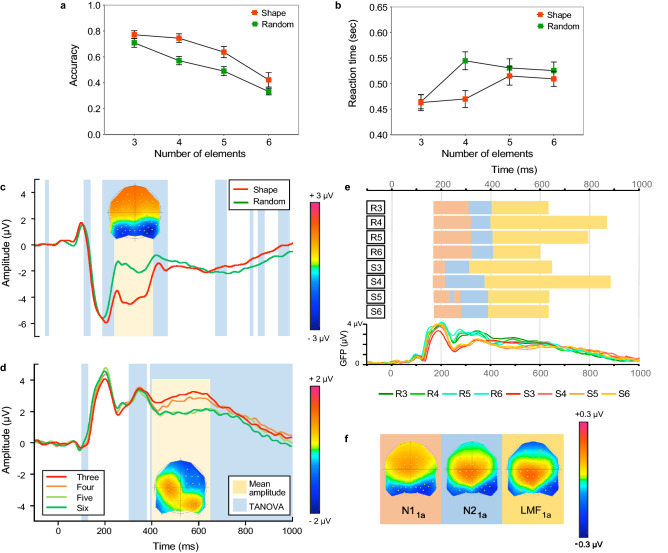
Results of Experiment 1. Accuracy **(a)** and reaction times **(b)** as a function of number of elements when elements were located on the vertices of simple shapes (red) or random (green). **(c)** Grand average ERPs collapsed across number of elements, for shape (red) and random (green) configurations. The ERPs are an average of the six channels (P7, P8, PO5, PO6, PO7, & PO8) used to quantify the N1 and N2 components and highlights the N2 effect between 250 and 400 ms after stimulus onset (yellow region and difference topography between all shape and random conditions). Blue regions represent TANOVA differences between shape and random configuration stimuli. **(d)** Grand average ERPs collapsed across spatial configuration for different number of elements. The ERPs are an average of the LMF analysis electrodes (FC1, FC3, C1, and C3) between 400 and 650 ms (yellow region and difference topography). The difference topography, for graphical purposes, is the difference between low (3, 4) minus high (5, 6) number of elements irrespective of spatial configuration. TANOVA differences (blue regions) are also for low versus high number of elements. **(e)** Microstate segmentation of grand averaged ERPs for all eight conditions. Horizontally oriented bars show stable microstates and the point in time when they change for each condition (R3—random configuration with 3 elements, through to S6—shape configuration with 6 elements). Each map is represented by a different colour. Further, Global Field Power (GFP) waveforms, a measure of differences in the scalp electric field strength, are displayed for a visual comparison to topographic microstates which are independent of field strength. **(f)** Topographic microstates derived from the segmentation procedure for three maps which best fit the individual subject data. Topographic maps show the head from above with nasion plotted upward.

Figures 2 shows accuracy (Fig. 2a) and reaction times (Fig. 2b) as a function of the number of elements when located on the vertices of simple shapes (red symbols) or random (green symbols). The results indicate significantly faster RTs and higher accuracy when the configuration of elements was shape-like rather than random (compare red and green symbols). Further, RTs increased and accuracy decreased as the number of elements increased.

A two-way repeated measures ANOVA with factors spatial configuration (shape vs. random) and number of elements (3, 4, 5, 6) revealed a significant main effect of spatial configuration (F(1,18) = 78.332, *p* < 0.0001, η^2^ = 0.813 for accuracy; F(1,18) = 27.289, *p* < 0.0001, η^2^ = 0.603 for RTs) with faster RT and higher accuracy for shape compared to random configuration. The effect of number of elements (F(3,54) = 49.351, *p* < 0.0001, η^2^ = 0.733 for accuracy; F(3,54) = 23.608, *p* < 0.0001, η^2^ = 0.567 for RTs) was also significant. In addition, a significant interaction effect for RTs (F(3,54) = 23.019, *p* < 0.0001, η^2^ = 0.561) but not for accuracy (F(3,54) = 2.023, *p* = 0.122, η^2^ = 0.101) was found. Bonferroni-corrected post-hoc analysis on the RTs data was carried out to examine the significant interaction effect. This revealed that all pairwise comparisons were statistically significant (*p* < 0.0001) except comparisons between 3 and 4 elements and between 5 and 6 elements, irrespective of their spatial configuration. Thus, RT data was shown to separate low from high numeric quantities (3, 4 vs. 5, 6) irrespective of their spatial configuration. Overall, these results suggest that shape configurations facilitate number estimation.

#### ERP analysis

The ERPs averages across number of elements for the shape and random configurations are shown in Fig. 2c and separated by number in Fig. S1a (see Supplementary Information). The ERPs corresponding to different number of elements, averaged across spatial configuration, are shown in Fig. 2d, and separated for each condition in Fig. S1b (see Supplementary Information). These results indicate a larger negativity between ~ 200 and 400 ms for random than shape configurations (compare red and green lines in Fig. 2c).

A repeated-measures ANOVA model with factors spatial configuration (shape vs. random), number of elements (3, 4, 5, 6), hemisphere (left vs. right electrode locations) and electrode (P7, PO5, PO7 vs. P8, PO6, PO8) carried out on the *N1 component* (160–220 ms) revealed that mean N1 amplitude increased with shape-like configurations in comparison to random (F(1,18) = 8.869, *p* = 0.008, η^2^ = 0.33) and with the number of elements (F(3,54) = 4.833, *p* = 0.01, η^2^ = 0.212). There was also a significant main effect of hemisphere (F(1,18) = 12.907, *p* = 0.002, η^2^ = 0.418), showing that N1 mean amplitude was largest over right occipital/parietal locations. This location difference appears to be driven in part by the larger amplitudes for shape configuration in the right hemisphere (F(1,18) = 4.872, *p* = 0.041, η^2^ = 0.213). Finally, there was a significant interaction between the configuration and number of elements (F(3,54) = 4.638, *p* = 0.014, η^2^ = 0.205). Bonferroni-corrected post-hoc analysis revealed that this interaction effect was driven predominantly by the large increase in N1 amplitude for three elements placed in a triangle compared to random configuration (*p* < 0.0001), with no other multiple-comparisons being statistically significant (*p* > 0.05). *In sum*, the N1 amplitude, which was maximal in the right hemisphere, was increased by shape configurations, and also by the least number of elements in the stimulus.

For the later *N2 component *(250–400 ms), a significant main effect of configuration was found (F(1,18) = 105.655, *p* < 0.001, η^2^ = 0.854) indicating that shape configuration elicits increased N2 amplitudes in comparison to random configurations (Fig. 2c; also Fig. S1a in the Supplementary Information). This was accompanied by a small increase in N2 amplitude with the number of elements (F(3,54) = 4.36, *p* = 0.031, η^2^ = 0.195)—see Fig. S1a in the Supplementary Information. There was no significant interaction effect between spatial configuration and number of elements (F(3,54) = 2.161, *p* = 0.128, η^2^ = 0.107). Like the N1 component, N2 amplitude was also maximal in the right hemisphere (F(1,18) = 7.218, *p* = 0.015, η^2^ = 0.286). This hemisphere effect interacted with spatial configuration suggesting a larger increase in amplitude for shape configurations in the right compared to the left hemisphere electrodes (F(1,18) = 5.554, *p* = 0.03, η^2^ = 0.226). Finally, we found a borderline three-way interaction between configuration, number of elements and hemisphere (F(3,54) = 2.828, *p* = 0.049, η^2^ = 0.136), which appears to be driven by a statistically significant effect of the randomly distributed elements in the left hemisphere sites only (*p* = 0.011).

The ANOVA model for the *LMF component* (400–650 ms) consisted of the factors configuration (shape vs. random), elements (3, 4, 5, 6) and electrode (FC1, FC3, C1, C3). Again, shape-like configurations produced larger amplitudes than random (F(1,18) = 15.599, *p* = 0.0001, η^2^ = 0.464), and the LMF appeared to separate the number of elements in the stimulus (F(3,54) = 23.03, *p* < 0.001, η^2^ = 0.561), with greater LMF amplitude observed for stimuli containing a low number in comparison to high number of elements (Fig. 2d; also Fig. S1b in the Supplementary Information). Post-hoc analyses revealed that all differences between number of elements were significant (all *p*’s < 0.05) except, interestingly, between stimuli with 5 or 6 elements (*p* > 0.05) which is consistent with the RT data. No other significant effects were found.

#### Topographical analysis and microstate segmentation

In order to map the time course of ERP differences that remain stable over time, we conducted paired TANOVA comparisons between all shape and random configurations, averaged across the number of elements (see light blue areas in Fig. 2c). In contrast blue areas in Fig. 2d shows TANOVA differences between low (3, 4) and high (5, 6) numeric quantities, averaged across shape configuration. TANOVA results suggest a large period of topographic differences for shape versus random configurations between 194 and 464 ms. For assessing number of elements we grouped conditions on low (3 and 4) versus high (5 and 6) numeric quantities, irrespective of their spatial configuration. This analysis revealed differences from 301 to 384 ms, and continuing at 392 ms until the end of the epoch. Importantly, TANOVA results incorporated the time windows identified for ERP mean amplitude analysis of the N2 (250–400 ms) for shape configurations and the LMF (400–650 ms) for number of elements. These TANOVA results suggest topographic differences that incorporate the time range of the N2 and LMF components, but critically, do not dissociate between the different topographic states.

To further clarify the observed TANOVA differences and their relation to shape configuration and number of elements, we ran a microstate segmentation analysis across the grand-averaged ERPs for all eight conditions of Experiment 1 (Fig. 2e,f). In terms of global explained variance (GEV), over the N1 time window the presence of one map (which we term N1_1a_) dominated the segmentation. GEV for N1_1a_ was subjected to a repeated measures ANOVA of spatial configuration (shape vs. random) and number of elements (3, 4, 5, 6). The N1_1a_ map best fitted conditions with a low number of elements (F(3,54) = 4.166, *p* = 0.019, η^2^ = 0.188), yet post-hoc analysis of the number of elements revealed no significant differences between pairs of elements (all *p*’s > 0.05). Thus, the N1_1a_ map was not related to changes in shape configuration or the number of elements, implying that processes in the N1 time window are generic for all conditions of the experiment. Considering that TANOVA fails to find topographic differences in the N1 range, it is of no surprise that the only observable difference in N1 topography is due to electric field strength rather than differences in topographic distribution.

Over the N2 time window, three maps were clearly observable: the N1_1a_ map and two other maps which we label N2_1a_ and LMF_1a_ respectively (Fig. 2e,f). When Map is included as a factor in the ANOVA model, no difference was observed between all three maps (N1_1a_, N2_1a_, LMF_1a_). There was a main effect of configuration such that each map explained more variance for shape configurations compared to random (F(1,18) = 21.634, *p* < 0.001, η^2^ = 0.546). Importantly, a significant map by shape interaction was found (F(2,36) = 12.213, *p* < 0.001, η^2^ = 0.404), and further investigation revealed that the shape versus random configuration effect is driven by the N1_1a_ map predominantly (F(1,18) = 23.807, *p* < 0.0001, η^2^ = 0.569), with this map explaining more variance for shape compared to random, while the N2_1a_ map was insensitive to spatial configuration (F(1,18) = 0.672, *p* = 0.423, η^2^ = 0.036). Hence the N1_1a_ map responds preferentially to shape configurations in the N2 time window. Note that the presence of the N1_1a_ map in the N2 time window is not unusual, but reflects the labelling of each microstate by the first point in time when they occur in relation to ERP time windows (see “Methods”). Finally, in the time window for analysis of the LMF (400–650 ms), both N2_1a_ and LMF_1a_ maps were present and analysed accordingly. Critically, N2_1a_ was found to be insensitive to the number of elements and also shape-like configurations (all *p*’s > 0.05). The LMF_1a_ map however, dissociated between the number of elements in the stimulus, thus explaining more variance in individual participant data for conditions with a low number of elements compared to high (F(3,54) = 3.472, *p* = 0.026, η^2^ = 0.162).

*In sum,* the microstate segmentation findings show that microstates N1_1a_ in the N2 time window (250–400 ms) indexed *the strength* of the shape versus random configuration effect, while the LMF_1a_ map dissociates between the number of elements in the stimuli.

### Effect of number of elements for a triangle shape configuration

**Figure 3:**
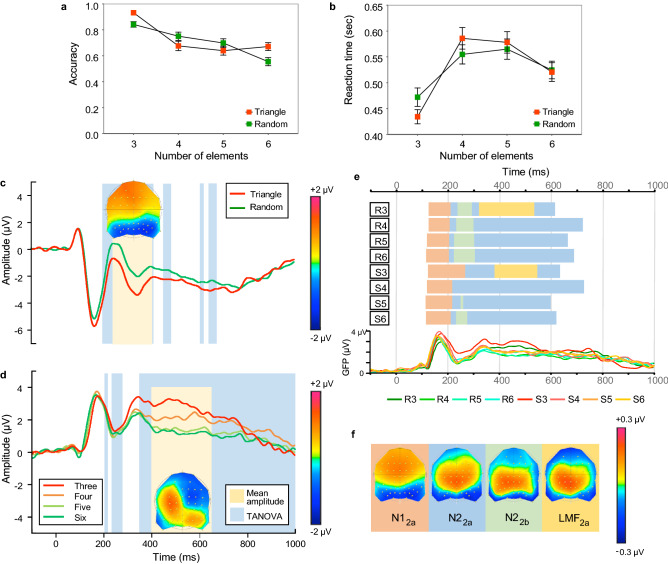
Results of Experiment 2. Accuracy **(a)** and reaction times **(b)** as a function of number of elements when elements were located on the vertices of an equilateral triangle (red) or random (green). **(c)** Grand average ERPs collapsed across number of elements for triangle (red) and random (green) configurations. The ERPs are an average of the six electrodes (P7, P8, PO5, PO6, PO7, PO8) used to quantify the N1 and N2 components and highlights the N2 effect analysed between 250 and 400 ms after stimulus onset (yellow region and difference topography between all shape and random conditions). Blue regions represent periods of stable topographic differences determined by the TANOVA. **(d)** Grand average ERPs collapsed across spatial configuration for different number of elements. The ERP waveforms, averaged across electrodes FC1, FC3, C1, and C3, display an effect of numeric quantity between 400 and 650 ms (yellow region and difference topography) over mid-left frontal electrode sites. The difference topography is the difference between low (3, 4) minus high (5, 6) number of elements, irrespective of spatial configuration. TANOVA (blue regions) also represent differences between low (3, 4) versus high (5, 6) number of elements. **(e)** Onset and offsets of topographic microstates for all eight conditions. Horizontally oriented bars show the point in time when microstate periods change for each condition of the experiment (R3—random configuration with 3 elements, through to S6—triangle configuration with 6 elements). Each map is represented by a different colour. Global Field Power waveforms are displayed for a visual comparison to topographic microstates. **(f)** Four topographic microstates derived from the segmentation procedure which best fit the individual subject data. Topographic maps show the head from above with nasion plotted upward.

We further investigated the effect of the number of shape vertices (or shape complexity) independent of the number of elements by utilizing the least complex shape (triangle) sampled by the full range of elements. Accuracy (Fig. 3a) and reaction times (Fig. 3b) are shown as a function of the number of elements for the triangle (red symbols) or random (green symbols) configurations.

A two-way repeated-measures ANOVA with factors spatial configuration (triangle vs. random) and number of elements (3, 4, 5, 6) revealed a significant main effect of number of elements (F(3,54) = 28.455, *p* < 0.0001, η^2^ = 0.613 for accuracy; F(3,54) = 34.627, *p* < 0.0001, η^2^ = 0.658 for RTs), suggesting that increasing the number of elements leads to overall reduced accuracy, and longer RTs. The effect of spatial configuration was not significant (F(1,18) = 4.398, *p* = 0.050, η^2^ = 0.196 for accuracy; F(1,18) = 0.023, *p* = 0.88, η^2^ = 0.001 for RTs). However, the interaction effect was significant for both accuracy (F(3,54) = 24.809, *p* < 0.0001, η^2^ = 0.580) and RTs (F(3,54) = 19.362, *p* < 0.0001, η^2^ = 0.518). Bonferroni-corrected post-hoc analysis on both accuracy and RTs data revealed that all pairwise comparisons were statistically significant (*p* < 0.0001), except comparisons between 4 and 5 elements, irrespective of their spatial configuration.

#### ERP analysis

The average ERPs obtained with triangle and random configurations are shown in Fig. 3c, and separated for each number of elements in Fig. S1c (see Supplementary Information). The ERPs corresponding to different number of elements, averaged across spatial configuration, are shown in Fig. 3d, and for each configuration in Fig. S1d (see Supplementary Information). As in Experiment 1, we analysed factors of configuration (triangle vs. random), number of elements (3, 4, 5, 6), hemisphere (left vs. right) and electrode (P7, PO5, PO7 vs. P8, PO6, PO8) for the N1 and the later N2 components.

The *N1 component* was increased in amplitude for triangle than random configurations (F(1,18) = 54.686, *p* < 0.001, η^2^ = 0.752), and also increased with the number of elements in the stimulus (F(3,54) = 4.677, *p* = 0.014, η^2^ = 0.206). Similar to Experiment 1, the largest N1 amplitudes were observed in the right hemisphere electrode sites (F(1,18) = 8.822, *p* = 0.008, η^2^ = 0.329). No interaction effects were found in the N1 range.

In the *N2 range*, triangle configurations elicited a larger negativity than random configurations (F(1,18) = 50.049, *p* < 0.001, η^2^ = 0.735). Like the N1 component, N2 amplitude was largest in right hemisphere electrodes (F(1,18) = 6.193, *p* = 0.023, η^2^ = 0.256). There was no significant main effect of the number of elements, however, there was an interaction between spatial configuration and number of elements (F(3,54) = 4.875, *p* = 0.009, η^2^ = 0.213), such that the largest N2 amplitudes were observed for triangle configurations with a low number of elements (compare red/orange lines with dark/light green lines in Fig. S1c in the Supplementary Information).

To investigate the LMF component (400–650 ms), we ran an ANOVA model with factors of spatial configuration (triangle vs. random), number of elements (3, 4, 5, 6) and electrode (FC1, FC3, C1, C3). The analysis revealed larger LMF mean amplitude for triangle configurations in comparison with the random conditions (F(1,18) = 11.013, *p* = 0.004, η^2^ = 0.38), and critically, the LMF also separated the number of elements in the stimulus (F(3,54) = 28.042, *p* < 0.001, η^2^ = 0.609), with an inverse relationship between LMF amplitude and number of elements (Fig. 3d; see also Fig. S1d in the Supplementary Information). This ordering by numerosity was evident in post-hoc analyses, with significant differences between all numbers of elements (all *p*’s < 0.05), except for 5 and 6 elements (*p* = 0.918). No other significant effects were found in the LMF range.

#### Topographical analysis and microstate segmentation

TANOVA analysis revealed topographic differences between triangle and random configurations, averaged across the number of elements, during the N2 time window (198 to 408 ms; blue shaded region in Fig. 3c). In contrast Fig. 3d shows TANOVA differences between low (3, 4) and high (5, 6) numeric quantities, averaged across shape configuration. This TANOVA analysis revealed a large sustained topographic difference between low and high number of elements starting at 335 ms and lasting until the end of the epoch, which again encompasses our analysis of the LMF (blue shaded region in Fig. 3d). These results suggest topographic differences that incorporate the time range of the N2 and LMF components, but critically, do not dissociate between the different topographic states.

Microstate segmentation analysis (Fig. 3e) revealed the presence of one map around the N1 range, which we term N1_2a_ (Fig. 3f). This map explained more variance, when fit to the individual participant data, for triangle compared to random configurations (F(1,18) = 19.05, *p* < 0.001, η^2^ = 0.514). This N1_2a_ map explained no other differences in individual participant data.

Over the N2 time-window, the presence of three maps was observed in the segmentation which were labelled as N1_2a_, N2_2a_, and N2_2b_ (Fig. 3e,f). We found a main effect of map (F(2,36) = 10.654, *p* = 0.003, η^2^ = 0.372) which revealed that the N2_2a_ map explained the least variance in comparison to N1_2a_ and N2_2b_ maps (post-hoc analyses showed all *p*’s < 0.02). No differences were found between N1_2a_ and N2_2b_ (*p* = 0.114). Although there was no significant main effect for triangle configurations, we found a significant interaction between map and configuration (F(2,36) = 6.768, *p* = 0.012, η^2^ = 0.273). Further analysis of this interaction showed that map N12a explains more variance in the data for triangle configurations compared to random conditions (F(1,18) = 9.471, *p* = 0.006, η^2^ = 0.345), whereas no significant effect of configuration for N2_2a_ and N2_2b_ could be found within the N2 time-window.

Segmentation analysis revealed two microstates present in the time window for analysis of the LMF, which we have termed the N2_2a_ and LMF_2a_ maps (Fig. 3f). Interestingly, while the presence of the LMF_2a_ map appears only in conditions containing three elements (which always produce either an equilateral triangle or scalene triangle), we found no differences in explanatory power of the two maps. In fact, we found a main effect of spatial configuration (F(1,18) = 7.497, *p* = 0.014, η^2^ = 0.294), suggesting that both maps better explain triangle than random configurations. There was no significant interaction effect between map type and spatial configuration. However, an interaction between map type and number of elements was found (F(3,54) = 7.076, *p* = 0.009, η^2^ = 0.282). Subsequent analysis revealed that the segmentation was dominated by the N2_2a_ map which extends from N2 time-window, and which explained most variance in individuals for triangle configurations (F(1,18) = 4.872, *p* = 0.041, η^2^ = 0.213). The LMF_2a_ map best fit conditions of 3 elements only (all *p*’s ≤ 0.011) in comparison to 4, 5 or 6 elements, irrespective of configuration (F(3,54) = 11.66, *p* = 0.001, ήp^2^ = 0.393). Thus, both N2_2a_ and LMF_2a_ maps better fit triangle than random configurations. No other significant effects were found in the segmentation analysis.

### Effect of luminance polarity on numerosity estimation

**Figure 4:**
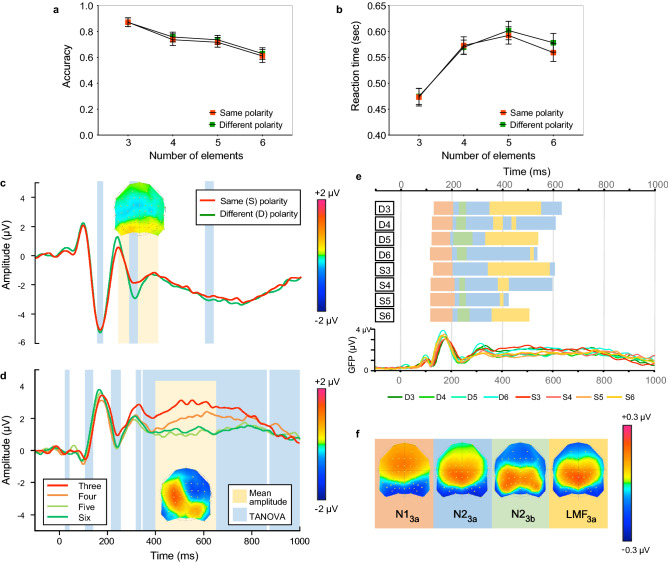
Results of Experiment 3. Accuracy **(a)** and reaction times **(b)** as a function of number of elements for same (red) and different (green) luminance polarity conditions. **(c)** Grand average ERPs collapsed across number of elements for same (red) and different (green) luminance-polarity conditions. The ERPs are an average of the six channels (P7, P8, PO5, PO6, PO7, & PO8) used to quantify the N1 and N2 components and highlights the N2 effect between 250 and 400 ms after stimulus onset (yellow region and difference topography between all same and different polarity conditions). The blue regions indicate periods of stable topographic differences determined by TANOVA. **(d)** Grand average ERPs collapsed across luminance polarity for different number of elements. These ERPs are an average over electrodes FC1, FC3, C1, and C3, and show the LMF between 400 and 650 ms (yellow region and difference topography). The scalp topography is the difference between low (3, 4) minus high (5, 6) number of elements. TANOVA (blue regions) are also for low (3, 4) versus high (5, 6) number of elements. **(e)** Topographic microstates of grand averaged ERPs for all eight conditions. Horizontally oriented bars show the microstates through time for each condition (D3—different polarity stimuli with 3 elements, through to S6—same polarity stimuli with 6 elements). Each map is represented by a different colour. Global Field Power waveforms are displayed for a visual comparison to topographic microstates. **(f)** Four topographic microstates derived from the segmentation procedure which best fit the individual subject data. Topographic maps show the head from above with nasion plotted upward.

Accuracy (Fig. 4a) and reaction time (Fig. 4b) data indicate only small differences between same and different luminance-polarity stimuli (compare red and green symbols in Fig. 4a,b). A two-way repeated measures ANOVA with factors luminance polarity (same vs. different) and number of elements (3, 4, 5, 6) revealed a significant main effect of luminance polarity (F(1,17) = 5.268, *p* = 0.035, η^2^ = 0.237 for accuracy; F(1,17) = 6.248, *p* = 0.023, η^2^ = 0.269 for RTs) and number of elements (F(3,51) = 14.154, *p* < 0.0001, η^2^ = 0.454 for accuracy; F(3,51) = 24.157, *p* < 0.0001, η^2^ = 0.587 for RTs). There was no significant interaction effect (F(3,51) = 0.356, *p* = 0.785, η^2^ = 0.021 for accuracy; F(3,51) = 2.669, *p* = 0.057, η^2^ = 0.136 for RTs). Further investigation into luminance polarity effects by comparing same and different polarity conditions for equal number of elements revealed that none of the multiple comparisons were significant (for accuracy: all *p*’s > 0.999; RTs: all *p*’s > 0.0733). In sum, we found small but significant mean differences between combined same versus different polarity conditions for accuracy (mean difference of 1.4%, SEM = 0.006) and RTs (mean difference of 7 ms, SEM = 0.003), most likely driven by the small variance around these combined mean differences, which becomes larger when split into individual conditions (smaller number of trials increases the calculated variance).

#### ERP analysis

The ERPs obtained with same and different polarity configurations are shown in Fig. 4c, averaged across number of elements, whereas ERPs corresponding to different number of elements conditions, irrespective of their polarity are shown in Fig. 4d. Separated waveforms for each number of elements conditions, averaged across electrodes used in the analysis of N1 and N2, and the LMF, respectively are shown in Fig. S1e,f in the Supplementary Information.

We ran an ANOVA model with factors luminance polarity (same vs. different), number of elements (3, 4, 5, 6), hemisphere (left vs. right electrode locations) and electrode (P7, PO5, PO7 vs. P8, PO6, PO8) for the N1 and the later N2 component. The analysis revealed no significant differences in the N1 range. In the N2 time-window of 250–400 ms, we found a significant effect of luminance polarity (F(1,17) = 8.52, *p* = 0.01, η^2^ = 0.334), with different luminance polarity stimuli eliciting an increased negativity (compare green and red lines in Fig. 4c, and Fig. S1e in the Supplementary Information). No other significant effects were found.

For the LMF component, the ANOVA analysis with factors luminance polarity (same vs. different), number of elements (3, 4, 5, 6) and electrode (FC1, FC3, C1, C3) revealed no effect of luminance polarity (F(1,17) = 2.93, *p* = 0.105, η^2^ = 0.147), but similar to Experiments 1 and 2, the LMF was modulated by the number of elements in the stimulus (F(3,51) = 26.948, *p* < 0.001, η^2^ = 0.613)—see Fig. 4d and Fig. S1f in the Supplementary Information. Furthermore, the modulation of the ERP amplitude by the number of elements appeared to change with luminance polarity (F(3,51) = 3.408, *p* = 0.037, η^2^ = 0.167). However, this interaction effect between number of elements and polarity appears to be caused mainly by 5 and 6 elements conditions. This effect cannot be generalised to luminance polarity changes, as it was not present in any other numerosity conditions. In sum, LMF amplitude was always largest for stimuli made of 3, then 4 elements, with 5 and 6 elements being almost equivalent (Fig. 4d; Fig. S1f in the Supplementary Information).

#### Topographical analysis and microstate segmentation

Paired TANOVA comparisons between all same and different luminance polarity conditions, averaged across the number of elements, are shown in Fig. 4c (see light blue areas). When examining the effect of luminance polarity with TANOVA three small but significant periods of topographic differences were observed: between 160 to 184 ms, between 294 and 326 ms and between 610 to 644 ms (see blue regions in Fig. 4c). The first of these periods encompasses the N1 peak, however, no ERP effect was found in the N1 range for polarity (see above), nor were there different microstates representing the change in polarity conditions in the N1 range (see Fig. 4c). The second significant period (294–326 ms), lies within the time range for N2 analysis, focused around the negative deflection observed for different luminance-polarity conditions. Finally, the period between 610 to 644 ms represents a later time-window that sits within the ERP analysis of the LMF. In contrast, when TANOVA was conducted on low (3, 4) versus high (5, 6) number of elements (blue areas in Fig. 4d), it revealed many differences, with the largest period of topographic difference starting at 325 ms and lasting until the end of the epoch, encompassing the analysis of the LMF component. These results suggest topographic differences for numeric quantity predominantly in the LMF time window, but critically, TANOVA does not dissociate between different topographic states.

Microstate segmentation revealed the presence of only one map, termed N1_3a_, in the N1 time range, yet subsequent ANOVA analysis revealed that this map was not specific to luminance polarity changes or the number of elements within the stimulus (Fig. 4e,f). However, there was an interaction effect between the luminance polarity and number of elements (F(2,34) = 4.791, *p* = 0.011, η^2^ = 0.21). Post-hoc analysis of this interaction found no significant differences between same and different polarity conditions, or between number of elements in the stimulus. Hence, we disregard this effect since the variance explained by map N13a differs based upon luminance polarity and the number of elements within the stimulus yet any differences between individual conditions are too small to be meaningful.

Over the N2 time-window, three maps were observed in the segmentation, which we labelled as N2_3a_, N2_3b_, and LMF_3a_ (Fig. 4f). Analysis of the variance in the individual data explained by these maps indicated differences between the three maps (F(2,34) = 4.55, *p* = 0.031, η^2^ = 0.211). Specifically, the N2_3a_ map fits the data in the N2 range better than the LMF_3a_ map (*p* = 0.04), but there were no differences between N2_3a_ and N2_3b_ (*p* = 0.184) or N2_3b_ and LMF_3a_ (*p* = 0.9999). The N2_3a_ map was the only map to fit the data better for different than same luminance-polarity conditions (F(1,17) = 4.69, *p* = 0.045, η^2^ = 0.216). Further, a main effect of the number of elements was found (F(3,51) = 3.018, *p* = 0.043, η^2^ = 0.151), however, no pairwise comparisons showed significant differences in the fit of the three maps according to the number of elements in the stimulus (all *p*’s > 0.05). This result indicates no graded pattern of response for any microstate in the N2 range based on numerosity.

Finally, two microstates were evident in the time window for analysis of the LMF, which we term the N2_3a_ and LMF_3a_ (Fig. 4f). Theoretically, each shift in microstate, irrespective of the reoccurrence of previous microstates, represents a change in underlying brain state. Both ${N2}_{3a}$ and ${LMF}_{3a}$, which are defined by the segmentation as different microstates, appear to be highly correlated with each other. Multiple shifts occur in the LMF time window between these maps. The analysis of the LMF time window revealed only a main effect of the number of elements explained by both maps (F(3,51) = 4.695, *p* = 0.015, η^2^ = 0.216) but no main effect of luminance polarity (*p* > 0.05). However, post-hoc analysis suggested that neither map could suitably explain the ordering of the number of elements in the LMF time window (all *p*’s > 0.05), since no map fitted a graded pattern of response from low to higher numeric quantities.

## Discussion

We aimed to systematically investigate the effect of shape configurations and luminance polarity of elements on numerosity estimation within and nearby the subitizing range using multiple analysis strategies. Across three experiments, our behavioural data show an effect of *shape configuration* on both RTs and accuracy (with faster RTs and higher accuracy for shape rather than random configurations), an effect of *number of elements* (with increasing numbers leading to overall reduced accuracy and longer RTs), and an effect of luminance polarity (with small differences in accuracy and RTs obtained with same and different luminance-polarity stimuli).

In terms of ERP and functional microstate data, we show across Experiments 1 and 2 that both N1 and N2 components, maximal in the right hemisphere, increase in amplitude for shape compared to random configurations. Interestingly, the N1 amplitude increase for numerosity did not follow an orderly, expected pattern, i.e., increasing from low to high number of elements (3, 4, 5, 6), as suggested by other studies^35–40,42^. This implies that the N1 component elicited with our stimuli, is not selective to numerosity per se (i.e., does not encode rank ordering of the number of elements). In Experiment 3, only the N2 component displayed an increased amplitude for different-polarity stimuli. Importantly, in all three experiments, the LMF increased in amplitude for stimuli containing a low number of elements (largest for 3 elements, then 4), but did not distinguish between higher numbers of elements in the stimuli (5 and 6 elements), nor was the LMF affected by luminance polarity. Note that our LMF responses cannot be driven simply by motor responses from participants, since participants made a response on every trial, and we included all trials in our analysis (see “Methods” section). Confirming our ERP findings, TANOVA analysis highlights topographic differences between shape and random configurations within the N2 range, and low and high number of elements in the LMF range (see blue regions in Fig. 2c,d; Fig. 3c,d; Fig. 4c,d). Furthermore, microstate segmentation confined numerosity estimation to a strict topographic distribution occurring within the LMF time window (maps ${LMF}_{1a}$ and ${LMF}_{2a}$), while a microstate responding to shape-configuration was evidenced earlier, in the N2 time window (maps ${N1}_{1a}$ and ${N1}_{2a}$; see Fig. 2e,f; Fig. 3e,f). The present results do not support the idea that shape coding and numerosity estimation within and nearby the subitizing range may be driven by the same neural mechanisms, rather, they suggest that number estimation follows shape coding and is facilitated by a shape-template matching process.

By showing temporally distinct ERP and microstate components that respond predominantly to shape configurations and number estimation, our data demonstrates that shape coding occurs before number estimation. Theoretically, this may imply that shape processing and subitizing are mediated by two separable independent mechanisms. However, we found that shape-configuration conditions also lead to an increase in amplitude of the LMF component in Experiment 1, and also in Experiment 2 only when the number of elements match the number of shape vertices. This implies that the processes supporting shape recognition and numerosity estimation interact with one another. Moreover, our behavioural data, indicating higher accuracy and faster RT for shape configurations, highlight the facilitation of number estimation mechanisms by shape template matching. In sum, facilitation of numerosity estimation by shape template matching, evident in our behavioural data, is reinforced by our ERP and microstate data.

Our conclusions are also supported by findings from other behavioural and ERP studies. While it has been suggested that number is processed independently of its visual properties^42,48^, a limited number of studies have argued against this idea^49–51^. Gebuis et al.^49–51^ showed that when visual cues were equated across stimulus displays, only N1 and P2 effects were present but no number-related effects occurred, thus, suggesting that number is estimated by weighting the different visual features/cues present in a stimulus. Previous ERP studies of subitizing have used stimuli presented in the *periphery* in a *variety of behavioural tasks* (e.g. feature-based attention, enumeration, visual search, working memory, match-to-sample tasks), and have identified two lateralized components specifically related to subitizing—the N2pc and CDA—for a review see^41^. The N2pc component is modulated by the number of perceived items, despite variations in stimulus properties, such as luminance change, size/area, total number of items in the stimulus^52–55^. In contrast, we found an N2 component that responded preferentially to spatial configuration, and also to luminance polarity changes, but did not consistently separate numerosity. Specifically, we found that the N2 was largest when the number of elements matched the number of shape vertices (Experiment 2), i.e. 3 elements on a triangle but not 4, 5, or 6 elements on a triangle. It may be tempting to interpret this finding as evidence for numerosity estimation and shape processing unfolding in parallel in the N2 range, however, we did not find direct evidence for this in our data. The ordering by number found in Experiment 2 occurred only when elements were presented in a shape configuration and not random, suggesting that N2 is sensitive to shape, and is agnostic to ordering of the number of elements. Given that we found the N2 to be modulated by visual features such as luminance polarity (Experiment 3) irrespective of feature-based attention, it is likely a perceptually-driven effect.

By comparing Experiments 1 and 2, one may wonder why the N2 component does not separate numerosity? The pattern of ERP data in the N2 range in Experiment 1 suggests that the N2 component increases in amplitude with larger numbers of elements, suggesting that there may be some earlier numerosity estimation responses than we report here, although comparisons were not significantly different (all *p*’s > 0.111). In Experiment 2, only an *equilateral* triangle was used as the shape configuration, which was sampled by an increasing number of elements (see Fig. 1c). However, a shape randomly-sampled by 3 elements will often make a *scalene* triangular shape, except when the 3 elements are placed by chance on the same side (or near the same side) of the virtual triangle (see Fig. 1b, leftmost panel). This configuration will often be perceived as a scalene triangle in both Experiments 1 and 2. Nevertheless, the effect of spatial configuration (equilateral vs. possible-scalene triangle) is still present in Experiment 2 only when the number of elements match the number of shape vertices. This is reflected in the pattern of behavioural response, ERPs, and may also be seen in microstate map ${LMF}_{2a}$ for shape versus random conditions. When the number of elements corresponds to the number of vertices, rather than separating number, the N2 component is increased in amplitude for shape configurations in comparison to randomly-placed elements.

Moreover, our data suggests that the N2 component, might also reflect a general processing of form or structure-like, as commonly observed in studies of symmetry perception^56,57^. These studies of symmetry have revealed a difference wave between ~ 250 and 600 ms termed the Sustained Posterior Negativity (SPN) that reportedly indexes symmetry perception and/or structure. SPN was found to be sensitive to luminance-polarity mismatch across the symmetry axis^57^, with early SPN differences driven not by symmetry sensitive mechanisms per se, but by pattern perception enhanced by the form/gestalt of the pattern (e.g., luminance-polarity grouping). In contrast to the symmetry literature, our N2 component is not as prolonged in duration (250–400 ms), yet this likely reflects subsequent numerosity estimation observed in the LMF time window. Similar to the symmetry literature, our N2 component was found to be sensitive to the luminance-polarity of the elements, with different luminance-polarity stimuli eliciting an increased negativity (Fig. 4c). Our ERP and behavioural results on the effect of luminance-polarity also complement previous behavioural findings showing selectivity to luminance polarity for contour-shape^31,34^ and texture-shape^58^ as well as other visual dimensions, e.g., illusory contour perception^59^. In line with the above-mentioned literature showing improved performance with stimuli that are consistent in luminance polarity (e.g., lower thresholds; larger aftereffects), our findings also show an improvement in performance as indicated by faster RTs and higher accuracy, in respect to different polarity conditions.

Our findings suggest that LMF overlaps the time course of the CDA found in previous studies. In feature-based attention studies of numerosity, the CDA component, which is a *sustained posterior negativity* occurring later (~ 300–400 ms after stimulus onset and lasting until the end of epoch) contralateral to the attended items, was found to increase monotonically with the number of items, and ostensibly represents visual working memory during periods of sustained attention^42,55^ (but note that no previous studies examining CDA used a working-memory task). In contrast, the LMF component amplitude is largest in response to low numeric quantities, supported by the presence of specific microstates within this range (see Experiments 1 and 2), whereas earlier microstates within N2 range restrict processing of the N2 ERP component explicitly to spatial configuration. Different from our N2 and LMF components, the previously defined N2pc and CDA are attention-mediated components generated by isolating targets from distractors via visual features (e.g. colour) that are elicited only under lateralized stimulus presentations. Our data extends upon these designs and clarifies the neural time course of numerosity estimation without interference from object individuation processes via feature-based attention.

The LMF component identified in the three experiments appears to separate numeric quantities and was not modulated by low-level visual features (e.g., luminance polarity). Do these LMF results, supported by behavioural data, imply that two separate processes for numerosity estimation are occurring for low (3, 4) and high (5, 6) numeric quantities, i.e., subitising (up to 4) compared to higher (5, 6) numerosity estimation? If two separable brain processes were activated for low versus high quantities, this would be evidenced by two distinct microstates, one corresponding only to low numeric quantities, and another for high. However, we found only a single microstate for all numerosities across the three experiments, suggesting that the same neural source generator is involved in processing all numeric quantities up to 6. That is, the processes involved in estimating low (3, 4) and high (5, 6) numeric quantities employ the same neural mechanisms, and they are not separated by a strictly-defined subitising limit of 4 proposed by some studies^4–6^, instead these processes may be maximally activated by low numeric quantities. In addition, one may be tempted to think that the processes indexed by the LMF are the same as those reflected in RT responses, i.e., that the LMF represents some form of post-perceptual processing, such as response monitoring. However, one should be cautious in inferring such a relationship given that response latency is only one of many possible consequences of stimulus presentation and it is not the end point of information processing^60^. It is highly likely that RTs and ERP component latencies are served by different subsets of processes invoked by stimulus presentation^60^. This is supported by numerous ERP studies showing that ERPs do not map directly with the behavioural task (for a few examples, see: Duncan-Johnson and Donchin^60^, Makin et al.^56^, Kuipers, Jones and Thierry^61^; Kutas and Federmeier^62^).

What brain processes underlie the ERPs in response to numerosity estimation? A number of possible processes may be indexed by any given signal. Several studies have suggested that in the context of higher numerosity, these processes may be an early unsegmented representation of the stimuli, a later refined/segmented representation, an abstract representation emerging in high-level areas, a decision process based on numerosity, and a working memory storage of such a representation^5,36,63–67^ . These suggested stages overlap roughly with those proposed by Mazza and Caramazza^41^. However, not all these stages are necessary for estimating number in the context of our study (low numerosity within the subitizing range), given that our elements were not embedded in a background and/or it was not required to segment a subset of elements from a background by virtue of feature-based attention (e.g., luminance polarity, colour, size). Although it is difficult to ascribe with certainty the contribution of each of these processes to a specific ERP modulation, our results demonstrate that the processes involved in shape coding occur before those responsible for quantifying number. Considering that we manipulated spatial configuration, while other studies have used only randomly distributed items^9,35–38^, it is highly likely that different processing steps were engaged when coding numerosity and spatial relationships (e.g., template matching) versus visual features (e.g., colour, luminance polarity), and these might take precedence over numerosity in this context.

In sum, our data reveals the neural time-course of numerosity estimation, which we replicated across three different participant samples: the LMF component, and its corresponding microstate, separated low numerical quantities up to 4 elements, but showed comparable responses for 5 or 6 elements. Furthermore, we show that number estimation can be facilitated when the number of elements in the stimulus matches the number of shape vertices, which was consistently observed in both behavioural data and ERPs (as indicated by the increase in amplitude of the LMF for shape configurations). It is interesting to note that luminance polarity increased N2 amplitude only, suggesting that shape but not numerosity estimation is selective to luminance polarity. Across all three experiments, the quicker RTs, higher accuracy, and increased LMF amplitude demonstrate that shape coding precedes number estimation, implying that subitizing can be facilitated by a shape-template matching process which considers the relationship between vertices.

## Methods

### Participants

A total of seventy-two observers (24 participants in each experiment), who were naive with regard to the experimental aims participated in this study. All observers had normal or corrected-to-normal visual acuity. Observers gave their written informed consent prior to participating and were treated in accordance with the Declaration of Helsinki (2008, version 6). All research procedures were approved by the University of Stirling Ethics Committee.

### Stimuli: generation and display

The stimuli were created using Matlab and presented on a Sony Trinitron monitor with a 1,024 × 768 spatial resolution and a refresh rate of 120 Hz. The R (red), G (green) and B (blue) outputs of the monitor were gamma-corrected after calibration with an Optical OP200E photometer. All stimuli were presented in the center of the monitor on a mid-gray background with mean luminance of 65.5 cd/m^2^. Viewing distance was 100 cm.

The stimuli consisted of a small number of elements, either 3, 4, 5 or 6 elements presented in the center of the monitor within a circular area of 8 deg diameter on the mean-luminance background (Fig. 1). The elements were Gaussian blobs with a standard deviation of 0.08 deg, a Gaussian size standard deviation factor of 5 and a contrast of 0.90. To avoid spatial overlap, the minimum distance between the elements was twice their size. In three different experiments, we varied the spatial configuration and luminance-polarity of the elements. To examine the effect of spatial configuration (Experiment 1), the elements were positioned either on the vertices (i.e. points of maximum curvature) of simple shapes, e.g., equilateral triangle, square, pentagons and hexagon (Fig. 1a) or randomly (Fig. 1b). All random conditions in each experiment refer to elements placed anywhere on the virtual contour-path of a shape, except its vertices. This was done in order to keep the size of the stimulus comparable across the shape and random conditions. Note also that in this experiment the number of elements and shape vertices were matched (i.e. three elements placed on the vertices of a triangle, four elements placed on the vertices of a square and so on—see Fig. 1a). Given the four shapes (triangle, square, pentagon and hexagon) and two spatial configurations (on-vertices vs. random), this resulted in eight stimulus conditions.

In Experiment 2, we dissociated between the number of elements and shape vertices by using only the simplest shape configuration—an equilateral triangle, which was sampled by either 3, 4, 5 or 6 elements, with three elements always positioned on the vertices and the remaining elements placed randomly anywhere within and including the virtual contour-path (Fig. 1c). These triangular shape conditions were compared with their corresponding random conditions.

Finally, to examine the effect of luminance polarity (Experiment 3) we used stimuli made of either same (all white or all black, Fig. 1c) or different (white and black, Fig. 1d) luminance-polarity elements with three elements always positioned on the vertices of a triangle and the remaining elements placed randomly anywhere within and including the virtual contour-path (as in Experiment 2). Given the two luminance-polarity conditions (same vs. different) and four number of element conditions (3, 4, 5, 6), this experiment resulted in eight stimulus conditions. In all experiments and conditions, the orientation of each virtual shape configuration was randomized from trial to trial.

### Procedure: reaction times and accuracy

Each experimental session started with a fixation cross followed by a stimulus presented for 100 ms and a uniform mid-gray background for 1,000 ms. In each experiment, there were eight stimulus conditions, with each condition presented 100 times, in random order, resulting in a total of 800 trials. The task for the observer was to indicate as quickly and as accurately as possible the number of elements perceived on the screen by pressing the corresponding key (e.g., ‘3’ for three elements, ‘4’ for four elements with the middle and index fingers of the left hand respectively, and ‘5’ for five elements and ‘6’ for six elements with the index and middle fingers of the right hand). We asked participants to use both their hands while responding in order to balance differences in motor responses within and between participants. No feedback was given to participants after responding. For each stimulus condition and observer, we measured accuracy (proportion correct answers) and reaction times (RTs) from the point of stimulus onset. We then calculated the average across-participants for each of these measures and the standard error across participants.

### Procedure: EEG recording and ERP analysis

For all three experiments, raw EEG signals were recorded from the scalp at a 1 kHz sampling rate from 64 Ag/AgCl electrodes distributed according to the extended 10–20 system and using CZ as the online reference. All electrode recording impedances were kept below 5 KΩ. The electroencephalogram was filtered on-line between 0.01 and 200 Hz and off-line with a band-pass zero phase shift digital filter between 0.1 and 30 Hz (12 db/octave and 48 db/octave slope, respectively). Eye blink artefacts were mathematically corrected using a model blink artefact computed for each individual based upon the method of Gratton, Coles and Donchin^68^. Signals exceeding ± 75 μV in any given epoch were automatically discarded. EEG recordings were cut into epochs ranging from − 100 ms to 1,000 ms after stimulus onset and averaged for each individual according to the experimental conditions. Grand-averages for each experiment were calculated after re-referencing individual participant ERPs to the common average reference. Participants whose data showed irretrievable noise contamination or a significant loss of channels, leading to no discernible ERP signal, were removed from the analysis. This resulted in a total of 19 participants in both Experiments 1 and 2, and 18 in Experiment 3 being used in the final analysis.

ERPs across all three experiments displayed a typical P1–N1–P2–N2 complex. The N1 component was identified as the first negative peak observable in waveform data and was maximal over posterior occipital electrode locations (P7, P8, PO5, PO6, PO7 and PO8), defined by topographic differences in the N1 range. Mean amplitude analysis of the N1 component was conducted between 160 and 220 ms. In addition, we analysed the negative component N2, occurring after the N1 in the time window 250 to 400 ms using the same electrode locations. Finally, we also identified a later component, which we termed Left-Mid-Frontal effect (or LMF), appearing to be modulated by the number of elements, and being maximal in the left hemisphere frontal/central electrode locations (FC1, FC3, C1, and C3 electrode locations) between 400 and 650 ms. These three components (N1, N2, and LMF) were observed across all three experiments and, for consistency, the same analysis approach (i.e. components, time windows, and electrode sites) was applied across all three data sets. All ERP data were subjected to repeated-measures ANOVAs, with each model explained fully in the results section for each experiment. Greenhouse–Geisser corrections were used where applicable. To demonstrate the magnitude of effects, partial eta-square (η^2^) is also reported. It is important to note that effects of numerosity, i.e., number of elements, found in ANOVA models may not necessarily mean that a given ERP component displays gradual changes in amplitude in line with increasing (or decreasing) numerosity. We consider an ERP component to be selective to number when a rank ordering of the number of elements is present in the statistical analysis, rather than randomly ordered differences.

### Procedure: topographic analysis and functional microstates

For each experiment, EEG data were subjected to further topographical analyses to look for stable patterns of scalp activity, which were performed using Cartool software^69^, brainmapping.unige.ch/cartool. The topography of the scalp potential field contains periods of quasi-stability for brief windows of time in which the strength of the electric field may vary but the field configuration remains stable^70–72^. Traditional waveform analysis of the EEG/ERP signal characterizes peaks and troughs of waveforms as components assumed to reflect different functional states of the brain, yet this approach is constrained by the choice of reference electrode, and can therefore lead to misinterpretations of data that can conflate field strength differences with a topographic configuration difference^73,74^. Since the configuration of the electric field at the scalp is independent of the choice of reference electrode, it can be assumed that changes in topography reflect underlying changes in neural source generators^70^.

We conducted paired topographic ANOVA (or TANOVA) comparisons for differences between conditions to assess changes in global dissimilarity (DISS). DISS is an index of configuration divergence between two electric fields over time, independent of their strength^75,76^. This analysis provides an objective measure of stable topographic differences by re-assessing single-subject maps to different experimental conditions—a non-parametric randomization test over each time point and all electrodes (we discarded periods of stability of less than 10 ms in duration). We use this method to highlight global trends in the ERP data. However, TANOVA can only identify when in time those differences arise, and not how those differences are generated. To highlight differences in topographic periods of stability not covered by TANOVA, we ran a microstate segmentation analysis^77^.

Using a hierarchical cluster analysis technique, we used grand averaged ERP data to produce a series of microstates in the form of topographic maps. The optimal number of microstates was found using a cross-validation criterion^77–79^, to determine the microstates that explain the greatest amount of variance in the ERP map series. Next, we assessed the statistical validity of our segmentation by determining the amount of variance explained in each map in the ERPs of each observer by condition for the time windows used in analysis of the three components: N1, N2, and LMF. For clarity, the naming of each microstate derived from the segmentation procedure relates to the overlap between each microstate and these ERP components, and is subscripted with the experiment number (1, 2, 3) and the order of microstate occurrence (a, b), e.g., N1_1a_, N1_1b_, N1_2a_ etc. Repeated-measures ANOVAs were carried out on the Global Explained Variance (GEV) of each microstate fitted to individual participant data, in order to compare how each map could explain each condition. This procedure was completed for each of the three experiments.

## Supplementary information

Supplementary information

## Data Availability

All behavioural and EEG data are available online: https://hdl.handle.net/11667/143.
