# Supplementary material for: Shape facilitates number: brain potentials and microstates reveal the interplay between shape and numerosity in human vision
Source: Sci Rep. 2020 Jul 24;10:12413. doi: 10.1038/s41598-020-68788-4 (PMC7381628; doi:10.1038/s41598-020-68788-4)
Supplement: Supplementary file 1 — Supplementary information [file 41598_2020_68788_MOESM1_ESM.pdf]

Supplementary Information

Shape facilitates number: brain potentials and microstates reveal the interplay between shape and numerosity in human vision

Elena Gheorghiu[1,✉] & Benjamin R Dering[1]

[1] *University of Stirling, Department of Psychology, Stirling, FK9 4LA, Scotland, United Kingdom*

✉ elena.gheorghiu@stir.ac.uk

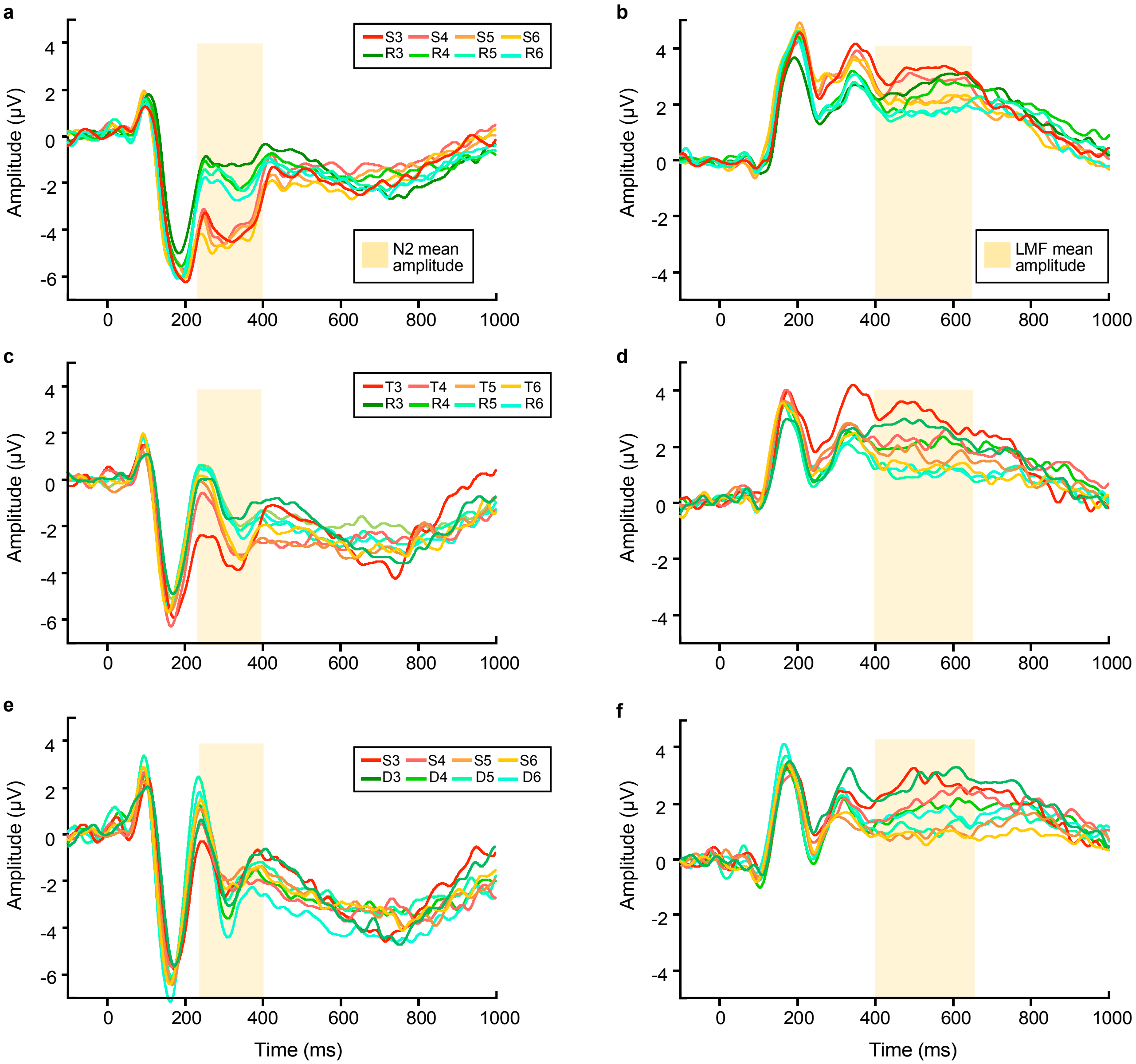

Fig.S1. *Results of all experiments.* *ERPs **(a,c,e)** are averages of the channels (P7, P8, PO5, PO6, PO7, & PO8) used to quantify the N1 and N2 components measured between 250-400ms (yellow region), and **(b,d,f)** are averages of the channels (FC1, FC3, C1, & C3) for the LMF component measured between 400-650ms (yellow region). **(a,b)** Experiment 1: Grand average ERPs for shape (red and orange lines) and random (green to turquoise lines) configurations, separated by the number of elements. **(c,d)** Experiment 2: Grand average ERPs for triangle (red and orange lines) and random (green to turquoise lines) configurations, separated by the number of elements. **(e,f)** Experiment 3: Grand average ERPs for same (red and orange lines) and different (green to turquoise lines) luminance polarity, separated by the number of elements. In all panels increasing numeric quantities are represented by lighter shades.*
